# An increase in dietary n-3 fatty acids decreases a marker of bone resorption in humans

**DOI:** 10.1186/1475-2891-6-2

**Published:** 2007-01-16

**Authors:** Amy E Griel, Penny M Kris-Etherton, Kirsten F Hilpert, Guixiang Zhao, Sheila G West, Rebecca L Corwin

**Affiliations:** 1 Department of Nutritional Sciences, 126 S Henderson Bldg, The Pennsylvania State University, University Park, PA 16802, USA; 2 The Huck Institutes of the Life Sciences, 201 Life Sciences Bldg, The Pennsylvania State University, University Park, PA 16802, USA; 3 Department of Biobehavioral Health, 315 Health & Human Development East, The Pennsylvania State University, University Park, PA 16802, USA

## Abstract

Human, animal, and in vitro research indicates a beneficial effect of appropriate amounts of omega-3 (n-3) polyunsaturated fatty acids (PUFA) on bone health. This is the first controlled feeding study in humans to evaluate the effect of dietary plant-derived n-3 PUFA on bone turnover, assessed by serum concentrations of N-telopeptides (NTx) and bone-specific alkaline phosphatase (BSAP). Subjects (n = 23) consumed each diet for 6 weeks in a randomized, 3-period crossover design: 1) Average American Diet (AAD; [34% total fat, 13% saturated fatty acids (SFA), 13% monounsaturated fatty acids (MUFA), 9% PUFA (7.7% LA, 0.8% ALA)]), 2) Linoleic Acid Diet (LA; [37% total fat, 9% SFA, 12% MUFA, 16% PUFA (12.6% LA, 3.6% ALA)]), and 3) α-Linolenic Acid Diet (ALA; [38% total fat, 8% SFA, 12% MUFA, 17% PUFA (10.5% LA, 6.5% ALA)]). Walnuts and flaxseed oil were the predominant sources of ALA. NTx levels were significantly lower following the ALA diet (13.20 ± 1.21 nM BCE), relative to the AAD (15.59 ± 1.21 nM BCE) (p < 0.05). Mean NTx level following the LA diet was 13.80 ± 1.21 nM BCE. There was no change in levels of BSAP across the three diets. Concentrations of NTx were positively correlated with the pro-inflammatory cytokine TNFα for all three diets. The results indicate that plant sources of dietary n-3 PUFA may have a protective effect on bone metabolism via a decrease in bone resorption in the presence of consistent levels of bone formation.

## Background

Accumulating evidence indicates that dietary fats can influence bone health. The omega-3 (n-3) polyunsaturated fatty acids (PUFA), in particular, may be beneficial, as they have been shown to inhibit the activity of osteoclasts and enhance the activity of osteoblasts in animals [1,2]. Optimal quantities of n-3 PUFA, thus, appear to inhibit bone resorption and promote bone formation. In addition, the ratio of n-6 to n-3 fatty acids in the diet may be important. Lowering the dietary ratio of n-6/n-3 PUFA increased bone marrow cellularity [3] and bone strength [4] in animals. In contrast, other studies have indicated that dietary saturated fatty acids (SFA) may adversely affect bone health. Animal research [5] and epidemiologic data [6] indicate that diets rich in SFA reduce bone mineral density. Possible mechanisms that may account for the effects of dietary fatty acids on bone include alterations in prostaglandin production, lipid oxidation, calcium absorption, inflammatory processes and osteoblast differentiation [2,7-11].

Few studies have evaluated the relationship between dietary fats and bone status in humans. Epidemiological studies have shown inverse relationships between the dietary n-6/n-3 ratio and bone mineral density in older adults [12], as well as between dietary saturated fat and bone mineral density in men [6]. In contrast, a tendency toward a positive relationship between eicosapentaenoic acid (EPA; n-3) consumption and bone density has been reported [13]. Supplementation trials also indicate positive effects of n-3 fatty acids on bone health. Twelve months of EPA supplementation, for instance, increased a measure of bone density in postmenopausal women [13]. Other studies [14,15] provided oil supplements containing γ-linolenic acid (GLA) (n-6), EPA and docosahexanoic acid (DHA) (n-3) to women. When these supplements were consumed for 16 weeks serum markers indicative of bone formation increased [16]. When consumed for longer periods of time, the results were mixed in that n-3/GLA supplementation increased bone density, compared to a placebo containing SFA in one study [14]; however, in the other study, there was no n-3/GLA benefit compared to no supplemental fat [15]. It is likely that the lack of effect observed by Bassey et al. [15] was due to the large measurement error associated with total body bone mineral density measurements and the shorter study period (12 vs. 18 mos.). In contrast, the study design employed by Kruger et al [14] used more specific measures of bone density (spine and hip bone mineral density scans) to quantify changes over time.

A lack of effect of n-3 fatty acids on bone health also has been reported. In a recent study of 179 menopausal women no change in bone mineral density was observed following supplementation of 40 g flaxseed/d (9.1 g α-linolenic acid; ALA) for 12 months [17]. Similarly, in a randomized controlled trial of chronically inflamed (elevated levels of c-reactive protein) patients with Crohn's disease, supplementation of 2.7 g/d of EPA and DHA and antioxidants (vitamins A, C and E and selenium) elicited no changes in markers of bone resorption and bone formation, despite a significant increase in plasma levels of EPA, DHA and selenium [18]. Taken together, the epidemiologic and supplemental feeding data provide some suggestive evidence that dietary fatty acids, specifically n-3 fatty acids, can affect bone health in humans. By design, supplement studies typically do not involve control of the background diet, and it is possible that differences across studies could be explained by failure to control for other nutrients that affect bone. Therefore, controlled feeding trials are needed in order to convincingly demonstrate a causal relationship between fatty acids and bone health. Whereas there are some positive data with marine-derived n-3 fatty acids, there is little known about the role of plant-based n-3 fatty acids (ALA) in bone health.

The present investigation is the first controlled feeding study in humans to evaluate the effect of increasing dietary ALA and, thereby, decreasing the ratio of n-6/n-3 PUFA on bone health. Plant sources of ALA (walnuts and flax) were used. We recently showed that an ALA-rich diet inhibits vascular inflammation and endothelial activation in addition to having substantial lipid lowering effects [19]. A growing body of evidence indicates that many of the pathophysiological events associated with CVD also are associated with low bone density. Given the literature indicating effects of dietary fatty acids on bone health, as well as reported associations between cardiovascular disease and low bone density/osteoporosis [20], we sought to determine if plant-based n-3 PUFA also would be beneficial to bone health.

## Subjects and methods

### Subjects

The present study was part of a larger study [19] that was designed to evaluate the effects of dietary fatty acids on cardiovascular disease risk factors. All twenty-three individuals (20 males and 3 females) participated in both studies. Subjects were recruited via advertisements in the local newspaper and fliers distributed across the campus of the Pennsylvania State University. Subjects who met the initial criteria during a phone screen reported to the Metabolic Diet Study Center (MDSC) for anthropometric measurements and baseline blood sampling. The Institutional Review Board at the Pennsylvania State University approved the experimental protocol and all subjects provided written informed consent.

Baseline subject characteristics are shown in Table 1. Subjects were classified as overweight or obese, i.e., BMI between 25 and 35 kg/m^2^. In addition, subjects were moderately hypercholesterolemic [baseline serum total cholesterol (TC) between 5.17 and 6.21 mmol/L, LDL-cholesterol (LDL-C) between 40^th ^and 90^th ^percentiles (by NHANES III), with HDL-cholesterol (HDL-C) between 25^th ^and 75^th ^percentiles (by NHANES III)], and had serum triglyceride (TG) levels less than 3.95 mmol/L. Thus, subjects in the present study were quite representative of the population in the U.S. that is at high risk for cardiovascular disease. The three females were postmenopausal, and had not received hormone replacement therapy (HRT) for at least 6 months prior to the start of the study.

**Table 1:** Subject Characteristics

	Men	Women	All
N	20	3	23
Age (y)	48.6 ± 1.6	58.3 ± 2.7	49.3 ± 1.6
BMI (kg/m^2^)	28.0 ± 0.7	28.5 ± 2.4	28.1 ± 0.7
*Serum Lipids/Lipoproteins*			
Total Cholesterol (mmol/L)	5.74 ± 0.12	6.58 ± 0.28	5.85 ± 0.12
LDL-Cholesterol (mmol/L)	3.90 ± 0.11	4.53 ± 0.23	3.98 ± 0.11
HDL-Cholesterol (mmol/L)	1.12 ± 0.05	1.36 ± 0.11	1.16 ± 0.04
Triglycerides (mmol/L)	1.55 ± 0.18	1.51 ± 0.29	1.54 ± 0.16

### Study design

A randomized, double-blind, balanced order, three-period crossover design was employed. A Latin-Square protocol was used to randomize the subjects into a sequence of three experimental diets, which differed in their fatty acid composition. Diet periods lasted 6 weeks and were separated by an approximate 3-week compliance break during which subjects consumed their usual diet. The period of time that subjects consumed the diets (6 weeks) exceeded that for which alterations in serum NTx and BSAP have been reported in other studies [21,22].

Subjects consumed either breakfast or dinner at the diet center on Monday through Friday; all other meals were prepared and packed for offsite consumption at the subjects' home. Diet compliance was monitored by the staff of the diet center and by the review of daily and weekly monitoring forms. Subjects' body weights, usual activities and exercise levels were maintained throughout the course of the study.

### Experimental diets

Three test diets were used: an average American diet (AAD) that served as the control diet, and two high-PUFA diets (low in saturated fat and cholesterol) that had different amounts of linoleic acid [LA, C18:2 n-6, (LA Diet)] and α-linolenic acid [ALA, C18:3 n-3 (ALA Diet)]. For each of the three experimental diets, eight calorie levels (1800 – 3900 kcal) were developed to meet different energy needs of the subjects. Unit foods (muffins) containing the same macronutrient profile of each of the test diets were used during each diet period to provide incremental adjustments of 100 kcal/day as needed to maintain body weight. The nutrient composition of the three test diets, verified by chemical analyses, is reported in Table 2. All diets were nutritionally adequate and met 2/3 of the established Dietary Reference Intakes [23]. In addition, each of the three test diets met or exceeded dietary recommendations for both ALA and LA [24]. Total fat (~35% energy), carbohydrate (~50% energy) and protein (~15% energy) were kept as constant as possible across the three experimental diets. There were no differences across the 3 diets for calcium and vitamin D.

**Table 2:** Nutritional composition of the three experimental diets (based on 2400 kcal/d); determined from chemical assay

Nutrients	AAD	LA Diet	ALA Diet
CHO*	49.8	46.8	46.3
Protein*	15.7	16.1	16.1
Total Fat*	34.5	37.1 †	37.6 †
SFA*	12.7	8.5 †	8.2 †
MUFA*	13.2	12.2	12.3
PUFA*	8.7	16.4 †	17.2 †
LA*	7.7	12.6 †	10.5 †¶
ALA*	0.8	3.6 †	6.5 †¶
LA/ALA (n-6/n-3)	9.5/1	3.5/1 †	1.6/1 †¶
Cholesterol** (mg/d)	310.8	303.5	305.0
Calcium** (mg)	975.3	1030.9	1016.8
Vitamin D** (μg/IU)	4.704/188.145	4.667/186.708	4.658/186.302

*Values are % energy; **Calculated using Nutritionist V; AAD: Average American Diet

LA Diet: A diet high in PUFA and LA; ALA Diet: A diet high in PUFA and ALA

CHO: Carbohydrate; SFA: Saturated fatty acids; MUFA: Monounsaturated fatty acids;

PUFA: Polyunsaturated fatty acids; LA: Linoleic acid; ALA: α-linolenic acid

†: P < 0.05 compared with AAD for assayed data

¶: P < 0.05 compared with LA Diet for assayed data

The ratio of n-6/n-3 fatty acids for the LA and ALA diets were 3.5/1 and 1.6/1, respectively. The n-6/n-3 fatty acid ratio for the AAD was 9/1. The ratios differed primarily because of a marked increase in ALA in the LA and ALA diets; the quantity of LA was high for both of these diets although it was slightly lower on the ALA diet. Walnuts and walnut oil, which are particularly rich sources of both n-6 and n-3 PUFA, represented half of the total fat in the two high-PUFA diets. The daily consumption of walnuts and walnut oil was 37 g and 15 g, respectively, for the diet providing 2400 kcal/day. Sources of walnuts in the diet included walnut granola, honey walnut butter, walnut pesto, and plain walnuts as a snack. Flaxseed oil, ~20 g/day for the 2400 kcal/day diet, also was used to increase the ALA content of the ALA Diet. Dietary calcium and Vitamin D levels were calculated using the Nutritionist V database (N-Squared Computing, First DataBank Division, San Bruno, CA).

### Serum samples

Twelve hour fasting blood samples were taken by venipuncture on two consecutive days. Whole blood was centrifuged at 3000 rpm for 15 minutes at 4°C. Serum samples were aliquoted and stored at -80°C until the conclusion of the study when all samples were analyzed together. The serum fatty acid profile was determined using gas chromatography as described by Zhao et al [19]. In short, serum total lipids were extracted using a chloroform:methanol mixture (1:1, v:v) containing BHT (Sigma) and heptadecanoic acid (used as an internal standard, Nu-Chek-Prep); serum fatty acid composition was determined using gas chromatography. Percentage of individual fatty acids was calculated according to the peak areas relative to the total area (total fatty acid was set at 100%). Serum N-telopeptides of type I collagen (NTx) were measured using an enzyme-linked immunosorbent assay (ELISA) kit (Ostex International, Seattle); the intra-assay coefficient of variation was 5.5%. Serum NTx is regarded as a reliable indicator of bone resorption since it originates from proteolytic cleavage of bone collagen by osteoclasts, rather than by downstream degradative processes [25]. Furthermore, measures of serum NTx exhibit less individual variability than do those of urine [26,27]. Serum bone-specific alkaline phosphatase (BSAP) was determined using a chemiimmunoluminescent enzymatic assay (Quest Diagnostics, Chantilly, VA). BSAP provides a general index of bone formation, and a specific index of total osteoblast activity [28]. Serum TNF-α, IL-6, IL-4 and IL-1β levels were measured using ELISAs in the Cytokine Core Laboratory of the Pennsylvania State University General Clinical Research Center using protocols previously described [29].

### Statistical analyses

All statistical analyses were performed using SAS for WINDOWS, release 8.2 (SAS Institute, Cary, NC). Data are expressed as least squares mean ± standard error. Normality testing was conducted for each outcome variable. The Kolmogorov-Smirnov test was used to identify 2 individuals whose NTx levels fell outside of the normal distribution and were classified as outliers. The mixed models procedure (PROC MIXED) was used to test for effects of diet, order of diet presentation, period, and their interactions. Tukey-Kramer adjusted *P *values < 0.05 were used to determine whether the differences in NTx among diets were significant. The magnitude and direction of diet effects were also examined by analyzing change scores (experimental diets – AAD), with AAD values as covariates. Pearson correlation coefficients were used to evaluate the relationship between the levels of NTx and the log-transformed levels of TNFα, IL-6, IL-4, and IL-1β across all diets. In order to account for the repeated measurement of outcome variables across three diet periods, an average correlation for each diet was calculated and the significance of this pooled correlation was then tested using a repeated measures ANOVA with the subject variable as the repeated measure. All analyses were performed on log-transformed values; all means reported represent unadjusted means.

## Results

All subjects completed the study (Table 1) and serum fatty acid analysis (Table 3) revealed excellent dietary compliance [19]. Statistical analyses were first completed with all subjects (n = 23) and then on all male subjects (n = 20). Both sets of analyses yielded similar results, thus men and women were pooled for all analyses presented. Subjects maintained their baseline body weight throughout the study. NTx values for one male and one female subject were classified as significant outliers and excluded from the analyses, based on the Kolmogorov-Smirnov test. The final sample size was 21.

**Table 3:** Fatty acid profile (mol %) of serum total lipid in subjects on the three experimental diets (n = 21)

	AAD	LA Diet	ALA Diet
**SFA**	34.7 ± 0.6	33.0 ± 0.5*	33.5 ± 0.5
**MUFA**	20.0 ± 0.6	17.0 ± 0.6**‡**	17.9 ± 0.6**‡**
**PUFA**	45.5 ± 0.7	50.1 ± 0.7**‡**	48.7 ± 0.7**‡**
*n-6 PUFA*	42.0 ± 0.6	44.9 ± 0.6**‡**	42.1 ± 0.6**§**
*LA*	33.3 ± 0.5	37.1 ± 0.5**‡**	34.6 ± 0.5*§
*AA*	8.2 ± 0.4	7.5 ± 0.4**†**	7.3 ± 0.4**‡**
**n-3 PUFA**	3.4 ± 0.3	5.2 ± 0.3**‡**	6.6 ± 0.3**‡§**
*ALA*	0.8 ± 0.2	2.1 ± 0.2**‡**	3.1 ± 0.2**‡•**
*EPA*	0.5 ± 0.1	0.7 ± 0.1**‡**	1.2 ± 0.1**‡¶**
*DPA*	0.6 ± 0.04	0.7 ± 0.04	0.7 ± 0.04**‡¶**
*DHA*	1.6 ± 0.1	1.7 ± 0.1	1.5 ± 0.1
**SFA/UNSAT**	0.53 ± 0.01	0.49 ± 0.01**†**	0.50 ± 0.01*
**LA/ALA**	49.6 ± 2.3	19.3 ± 2.3**‡**	12.4 ± 2.3**‡¶**
**n-6/n-3**	12.7 ± 0.5	9.0 ± 0.5**‡**	6.8 ± 0.5**‡§**

SFA: Saturated fatty acids; MUFA: Monounsaturated fatty acids; PUFA: Polyunsaturated fatty acids; n-6: omega-6; LA: Linoleic acid; AA: Arachidonic acid; n-3: omega-3; ALA: α-linolenic acid; EPA: Eicosapentaenoic acid; DPA: Docosapentaenoic acid; DHA: Docosahexaenoic acid; UNSAT: total unsaturated fatty acids

*: P < 0.05, **†: **P < 0.01,**‡: **P < 0.001, compared with AAD

**¶ **P < 0.05,**•: **P < 0.01,**§ **P < 0.001 compared with LA Diet

**Figure 1:**
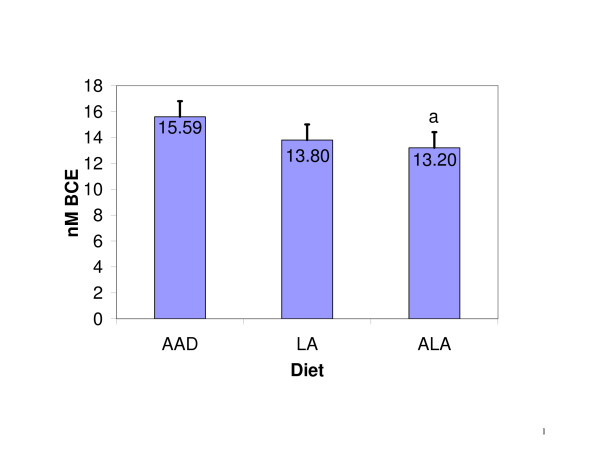
NTx levels when diets differing in fatty acid composition were consumed. ^a^p < 0.05 when compared to AAD. Vertical bars represent the standard errors of the means.

Mean NTx concentrations (± SE) at the conclusion of the three experimental diets were 15.59 ± 1.21, 13.80 ± 1.21, and 13.20 ± 1.21 nM BCE, for the AAD, LA and ALA diets, respectively (main effect of diet: p < 0.05). NTx levels were significantly lower following the ALA diet (p < 0.05) and there was a similar trend for the LA diet (p = 0.08), compared to the AAD diet (Figure 1). When change scores were analyzed with the control diet (AAD) NTx levels as a covariate, both the ALA and LA diets were associated with significant reductions in NTX concentrations (p < 0.05) relative to the AAD. The reduction following the ALA diet was 2.17 ± 0.68 nM BCE (15.3%), while the reduction following the LA diet was 1.77 ± 0.68 nM BCE (11.5%).

Levels of BSAP were unaffected by diet treatment. Mean concentrations of BSAP at the end of each diet period were 11.78 ± 0.80, 12.41 ± 0.80, and 11.94 ± 0.81 mcg/L, for the AAD, LA and ALA diets, respectively (p > 0.05). Similarly, the analysis of change scores adjusted for control diet BSAP showed no significant effects of the intervention diets.

The effects of these diets on serum cytokine levels are reported elsewhere [30]. In summary, levels of IL-6, IL-1β and IL-4 did not change after consumption of the three test diets. Levels of serum TNFα however were reduced following the ALA Diet (Median: 10.3 ng/L; Range: (1.2–12,072.7 ng/L), compared to the LA Diet (Median: 13.3 ng/L; Range: (1.2–1,513.0 ng/L) and the AAD (Median: 18.2 ng/L; Range: (1.2–1,232.5 ng/L) (main effect of diet: p < 0.08). In subsequent correlational analysis, data from the 16 individuals with detectable levels of TNFα were used. The levels of NTx were positively correlated with the levels of TNF-α(r = 0.54; p < 0.05), across all three diets. Thus, as TNF-α decreased with increasing intake of n-3 fatty acids, there were proportional decreases in NTx.

### Effects of diet on serum fatty acids

The serum fatty acid profile for subjects following the consumption of each of the three test diets is presented in Table 3. The changes observed in serum fatty acids validate our assessment of dietary compliance with daily and weekly monitoring forms. As expected, serum total n-6 PUFA were the highest on the LA diet (p < 0.001). Serum total n-3 PUFA, ALA, and EPA increased progressively across the LA and ALA diets, with the highest levels on the ALA diet. The ratios of serum SFA:UNSAT, LA:ALA and n-6/n-3 decreased significantly following the consumption of the LA and ALA diets, compared to the AAD (p < 0.001), and following consumption of the ALA diet compared to the LA diet (p < 0.001). There were no significant correlations between serum concentrations of fatty acids and BSAP or NTx.

## Discussion

This is the first controlled feeding study in humans to assess the effects of diets rich in ALA and LA provided by walnuts and flaxseed on bone health, as measured by serum NTx and BSAP, indicators of bone resorption and formation, respectively. We found that the high PUFA diets reduced serum concentrations of NTx, and maintained levels of BSAP relative to the consumption of a typical American diet, which is lower in PUFA and higher in SFA. NTx after consumption of the ALA diet in particular, was significantly lower than was NTx after consumption of the AAD. These effects are consistent with a reduction in bone turnover and maintenance of bone formation induced by relatively short-term consumption of the high PUFA diets. Although the effects of dietary fats on bone health have been studied in animals and through supplementation in humans, this is the first study to use a whole food source, incorporated into the diet of humans under controlled feeding conditions. Previous studies have shown beneficial effects of walnuts [19,31] and flaxseed [32] on CVD risk; the present results indicate potential benefits on bone health, as well.

Some scientists believe that the ratio of dietary n-6/n-3 PUFA is important to a variety of health outcomes [33]. The changes in n-6/n-3 ratios in the present study were accomplished by increasing the n-3 PUFA, while maintaining relatively constant levels of n-6 PUFA (Table 2). NTx levels were significantly lower when subjects consumed the ALA diet (n-6/n-3 ratio: 1.6) than when they consumed the AAD (n-6/n-3 ratio: 9.5); when subjects consumed the LA diet (n-6/n-3 ratio: 3.5), NTx levels were marginally significantly lower than when they consumed the AAD (p = 0.08). This stepwise reduction in NTx across the three diets (ALA < LA < AAD) indicates a possible dose-response effect of dietary n-3 fatty acids and/or reductions in the n-6/n-3 ratio on bone resorption. The stepwise nature of the results also indicates that the reductions in NTx were not due simply to the lower dietary SFA or the elevated LA in the LA and ALA diets.

The present results do not clearly distinguish if the observed effects are due to the ALA or to the conversion of ALA to EPA and its subsequent effects. The majority of studies investigating the effects of n-3 fatty acids on bone health have used fish oil, which is rich in EPA. However, a recent epidemiologic study showed that reduced LA/ALA ratios were associated with increased hip bone mineral density [12]. In the present study, plant sources of n-3 fatty acids were used; therefore, ALA was the primary n-3 fatty acid provided by the diet. In addition, the amount of ALA in the ALA diet (~17 g/day on the 2400 kcal diet) was greater than that previously provided in a supplementation trial (~9 g/day) [17]. Both serum ALA and EPA were significantly greater in the LA and ALA diet conditions than in the AAD condition. However, serum ALA increased to a greater degree than did EPA, which reflects the well established fact that little ALA is converted to EPA (~5–10%), and little is converted to DHA (< 1%) [34,35]. In the presence of a diet that is relatively high in n-6 PUFA (such as the LA and ALA diets in the present study), this conversion is reduced by 40 to 50% [36]. Therefore, it is possible that in the present study the results are reflective of an increase in ALA or due to potent effects of EPA. Further research is needed to clarify these possibilities.

The reduction in serum NTx that occurred in the present study following the consumption of the ALA diet for 6 weeks (15.3%) is somewhat less than reports after two weeks and 1 year of hormone replacement therapy (23% and 52%, respectively) or after 6 months of alendronate therapy (30.4%) [37,38]. Whether longer-term intake of n-3 PUFA would produce larger decreases in NTx and/or increases in bone density is not known. Regardless, the present findings confirm and extend a growing literature indicating beneficial effects of n-3 PUFA and reduced n-6/n-3 ratios on bone health.

The present results are consistent with animal work in which appropriate amounts of n-3 PUFA reduced osteoclast activity [1]. One mechanism that might account for this involves local alterations of fatty acid and prostaglandin concentrations within bone tissue [8]. Increased consumption of n-6 PUFA increases the ratio of arachidonic acid (AA) to EPA, and increases PGE_2 _concentration in bone [28]. Conversely, an increased consumption of n-3 PUFA decreases the AA:EPA ratio, and decreases PGE_2, _concentration and release from bone. This cascade of effects is potentially important, as PGE_2 _has been reported to stimulate bone resorption [see 10 for review]. EPA serves as a precursor for the formation of PGE_3_, which also can stimulate bone resorption. However, the conversion of EPA to PGE_3 _is much less efficient than is the conversion of AA to PGE_2 _[39]. Although measurements of bone prostaglandins were not taken in the present study, the serum fatty acid results showed that serum EPA was significantly greater on the ALA diet than on either the LA diet or the AAD [19], while AA was significantly reduced. It is possible that this shift in fatty acid synthesis decreased production of PGE_2 _within the bone while having minimal effect on PGE_3 _formation. The net result would be a reduction in osteoclast activity and serum NTx concentrations following the ALA diet.

We also examined the relationship between NTx and cytokines involved in bone remodeling, i.e. tumor necrosis factor-alpha (TNFα), IL-6, IL-4, and IL-1B [40,41]. We previously reported that TNF-α was significantly lower when the ALA diet was consumed relative to the AAD and LA diets [30]. In the present study, we showed that TNF-α levels were correlated with NTx levels across all of the diets. TNF-α promotes osteoclastic bone resorption and inhibits bone collagen synthesis in vitro [42], effects that may be mediated by PGE_2 _[43]. In addition, TNF-α expression was enhanced by AA in human osteoblast cells, an effect that was attenuated by the n-3 fatty acid EPA [44].

Although TNF-α promotes osteoclastogenesis via an autocrine mechanism [45], systemic TNF-α also has been shown to stimulate bone resorption, increase the number of circulating preosteoclasts [46,47], and elevate plasma calcium [43]. Furthermore, supplementation with flaxseed oil, which is a rich in ALA, reduced systemic TNF-α by about 30% in humans [48], as well as in wild-type and IL-10 knockout mice [49], and increased bone mineral content in IL-10 knockout mice [49]. Systemic TNF-α therefore, may be an important marker of, and/or contributor to, bone resorptive mechanisms. Taken together, the present results are consistent with literature indicating that dietary ALA can reduce bone resorption possibly through reduced production of TNF-α.

In contrast to the effects of the diets on a marker of bone resorption, there were no effects on a marker of bone formation (BSAP) in the present study. Although others have reported that low dietary n-6/n-3 ratios increased BSAP in developing rats, the present results are consistent with other negative reports in adults. For instance, no change in plasma alkaline phosphatase activity was seen in adult rats maintained on high n-3 diets [50]. In addition, no change in BSAP occurred when supplements containing a mixture of n-6 and n-3 fatty acids were given to adult women [14,15] over an extended period of time (≥ 12 mo), even though bone density increased in one of these trials [14]. The profile of effects reported here (reduced NTx, no change in BSAP) is similar to that reported for a variety of factors affecting bone health including dietary protein and sodium [51,52], nasal spray calcitonin (after 3 mo) [53], and calcium supplementation [54]. Such a profile suggests a reduction in bone turnover, and a shift in the balance of bone degradation/formation toward formation.

In the present study, most of the subjects were middle-aged men. While this population is generally overlooked in terms of bone health, the number of men who are afflicted with osteoporosis continues to grow [55]. Furthermore, recent epidemiologic data suggest that the effects of dietary fats on bone health may be particularly strong in men [6]. These reports as well as the present results indicate that bone health in men bears investigation, and that bone integrity in men, as well as in women, may benefit from manipulations of the dietary fatty acid profile.

In summary, the results of the present study indicate that incorporating walnuts and flaxseed into the diet as a means to increase ALA, and consequently decrease the n-6/n-3 ratio, reduces serum NTx and maintains levels of serum BSAP. The reductions in NTx were related to the amount of ALA each diet provided. Omega-3 fatty acids are known to have beneficial effects on the cardiovascular system, but few studies have examined their effects on human bone health. The present results suggest that incorporating plant sources of n-3 PUFA into the diet may provide health benefits not only to the cardiovascular system, but also to the skeletal system.
